# Modelling dynamical 3D electron diffraction intensities. II. The role of inelastic scattering

**DOI:** 10.1107/S2053273323010690

**Published:** 2024-01-25

**Authors:** Budhika Mendis

**Affiliations:** a Department of Physics, Durham University, South Road, Durham, DH1 3LE, United Kingdom; Helmholtz Centre for Infection Research, Germany

**Keywords:** inelastic diffuse scattering, phonons, plasmons, Bloch waves

## Abstract

The strong interaction of high-energy electrons with a crystal results in both dynamical elastic scattering and inelastic events, particularly phonon and plasmon excitation, which have relatively large cross sections. For accurate crystal structure refinement it is therefore important to uncover the impact of inelastic scattering on the Bragg beam intensities. Here a combined Bloch wave–Monte Carlo method is used to simulate phonon and plasmon scattering in crystals. The simulated thermal and plasmon diffuse scattering are consistent with experimental results. The simulations also confirm the empirical observation of a weaker unscattered beam intensity with increasing energy loss in the low-loss regime, while the Bragg-diffracted beam intensities do not change significantly. The beam intensities include the diffuse scattered background and have been normalized to adjust for the inelastic scattering cross section. It is speculated that the random azimuthal scattering angle during inelastic events transfers part of the unscattered beam intensity to the inner Bragg reflections. Inelastic scattering should not significantly influence crystal structure refinement, provided there are no artefacts from any background subtraction, since the relative intensity of the diffracted beams (which includes the diffuse scattering) remains approximately constant in the low energy loss regime.

## Abstract 2

An experimental and computational investigation is presented of the role of inelastic scattering on electron diffraction intensities.

## 1. Introduction

Three-dimensional electron diffraction (3D-ED) is a rapidly emerging technique for crystal structure characterization within a transmission electron microscope (Kolb *et al.*, 2007 ▸; Mugnaioli *et al.*, 2009 ▸; Zhang *et al.*, 2010 ▸; Nederlof *et al.*, 2013 ▸; Gemmi *et al.*, 2019 ▸). Since electrons are charged, it is possible to analyse nanometre-sized crystals that would otherwise be too small for X-ray diffraction. However, even for these relatively thin samples the Bragg-diffracted intensities must be treated dynamically for accurate results (Palatinus *et al.*, 2015*a* ▸; Klar *et al.*, 2023 ▸; Cleverley & Beanland, 2023 ▸). Despite the inevitable improvement in crystal structure refinement, the *R* factors for electron diffraction are still larger than the corresponding X-ray values (Klar *et al.*, 2023 ▸). The discrepancy could be due to a number of reasons, such as a non-uniform crystal shape (most calculations assume the specimen to be a parallel-sided slab), crystal mosaicity, extraneous surface layers and surface damage, or specimen bending. In all but the very thinnest samples, however, inelastic scattering will be omnipresent, particularly low-energy excitations such as thermal diffuse scattering (TDS) from phonons and plasmon scattering (Egerton, 1996 ▸). In general, energy filtering improves the crystal structure solution (Gemmi & Oleynikov, 2013 ▸; Eggeman *et al.*, 2013 ▸; Yang *et al.*, 2022 ▸), although there are also reports where no benefits were observed (Palatinus *et al.*, 2015*a* ▸). Latychevskaia & Abrahams (2019 ▸) have argued that inelastic scattering mitigates strong dynamical diffraction in protein crystals, so that useful crystal structure information can still be extracted from relatively thick specimens.

Bloch waves (Hirsch *et al.*, 1965 ▸; Spence & Zuo, 1992 ▸) are often used for simulating 3D-ED data (Palatinus *et al.*, 2015*b* ▸), but in its current form this approach is not particularly suitable for inelastic scattering. For example, TDS is modelled phenomenologically by introducing an imaginary term to the atom scattering factor, the so-called ‘absorptive form factor’ (Hall & Hirsch, 1965 ▸; Bird & King, 1990 ▸). This results in a depletion of intensity as the electron beam propagates through the crystal. The missing intensity is the TDS, and no details about its angular distribution, such as Kikuchi bands, can be obtained. The standard method for simulating TDS is therefore multislice (Cowley & Moodie, 1957 ▸; Kirkland, 2010 ▸) coupled with frozen phonons (Loane *et al.*, 1991 ▸). Recently, Monte Carlo methods have also been introduced to simulate plasmon scattering using multislice (Mendis, 2019 ▸; Barthel *et al.*, 2019 ▸; Mendis *et al.*, 2020 ▸; Mendis, 2023*a* ▸). Despite multislice being better at modelling inelastic scattering, it is not very convenient for simulating 3D-ED data sets. This is because of the need to create supercells that satisfy periodic boundary conditions, which is impossible to achieve in an electron diffraction tomography series. In principle, it is possible to minimize aliasing artefacts with large supercells and a Hanning window, although this increases the computing cost. The Bloch wave method, on the other hand, only requires the crystal unit cell as input and can therefore easily be adapted to different specimen orientations and/or beam incident angles (*e.g.* precession electron diffraction).

In this work, the Monte Carlo method of Mendis (2019 ▸) is combined with Bloch waves to simulate both phonon and plasmon inelastic scattering. In this new scheme there is no loss of electron intensity due to TDS, and all features in the diffraction pattern, including Kikuchi bands, are reproduced. Using both experiment and simulation, it is shown that inelastic scattering has an important effect on the relative intensities of Bragg reflections with respect to the unscattered beam. The paper is organized as follows. In Section 2 the theory behind Bloch wave inelastic scattering is presented. Section 3 outlines the experimental and simulation method­ologies, while results are presented in Section 4. The paper concludes with a discussion on the implications of inelastic scattering for crystal structure refinement (Section 5).

## 2. Bloch wave inelastic scattering

The Bloch wave solution for elastic scattering within a perfect crystal is (Spence & Zuo, 1992 ▸)

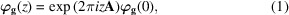

where **φ**_**g**_(*z*) is a column vector of Bragg-diffracted wavefunctions at depth *z* and **A** is the so-called structure matrix, whose elements are defined by

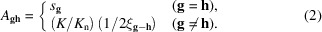


*s*_**g**_ and ξ_**g**_ are the deviation parameter and extinction distance, respectively, for the reciprocal vector **g**, while *K* is the incident electron wavenumber with component *K*_n_ along the specimen surface normal. Strictly speaking, the incident wavevector must be corrected for the mean inner potential of the crystal, although this has only a small effect on high-energy electron diffraction. The matrix exponential in equation (1) is conveniently evaluated by first performing an eigen decomposition of the structure matrix, *i.e.*


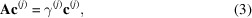

where **c** ^(*j*)^ is the eigenvector and γ^(*j*)^ the eigenvalue. Note that **A** is a *N*×*N* square matrix and **c** ^(*j*)^ is a *N*×1 column vector. There are *N* solutions of **c** ^(*j*)^ and γ^(*j*)^ that satisfy equation (3), which can be used to re-express equation (1) as

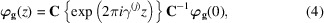

where **C** is a square matrix consisting of all eigenvector solutions **c** ^(*j*)^, and 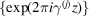 is a diagonal matrix with the *N* values of 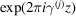 along its diagonal. Starting from the illumination conditions at the entrance surface, *i.e.* **φ**_**g**_(0), the elastically scattered Bragg beam intensities |**φ**_**g**_(*z*)|^2^ can be calculated at any given depth within the crystal using equation (4).

**Figure 1:**
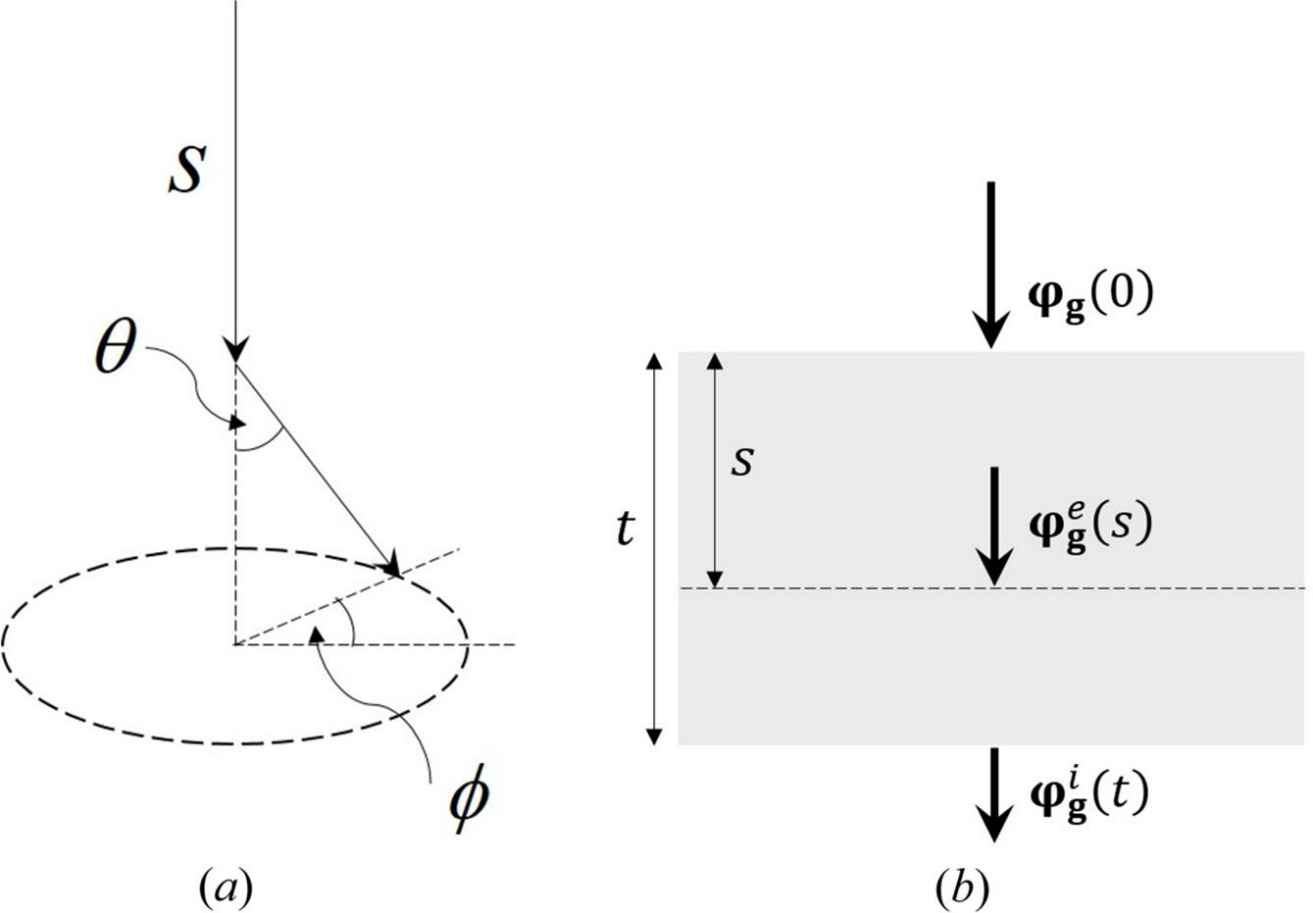
(*a*) A schematic diagram of the Monte Carlo simulation method. The incident electron travels a distance *s* before being inelastically scattered by polar angle θ and azimuthal angle ϕ. (*b*) An illustration of how the electron-beam wavefunction is altered by a single inelastic scattering event at a depth *s* in a specimen of thickness *t*. **φ**_**g**_(0) is the incident-beam wavefunction, 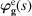 is the elastic scattered wavefunction at a depth *s* and 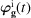 is the inelastic wavefunction at the specimen exit surface.

Next we consider inelastic scattering. It can be shown that, for highly delocalized excitations, such as low-momentum phonons and plasmons, the incident electrons effectively show particle-like behaviour (Mendis, 2020*a* ▸). Therefore, Monte Carlo methods can be applied to phonon and plasmon inelastic scattering. The principle is illustrated schematically in Fig. 1 ▸(*a*). The incident electron travels a distance *s* before undergoing inelastic scattering, which causes a deflection in the electron trajectory by polar and azimuthal angles θ and ϕ, respectively. The parameters *s*, θ and ϕ are estimated using computer-generated random numbers, in a manner that is consistent with the physics of the underlying scattering mechanism (Joy, 1995 ▸). As an example, for uncorrelated phonons (*i.e.* atoms vibrating independently of one another) and plasmons, there is no azimuthal dependence in the scattering, so that ϕ can be estimated using a uniform random number ( 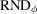 ) in the range [0, 1],

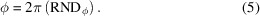

The scattering pathlength *s*, on the other hand, obeys a Poisson distribution with mean free path λ. To estimate *s* we first define a uniform random number (RND_*s*_) in the range [0, 1], which can also be expressed as (Mendis, 2019 ▸)

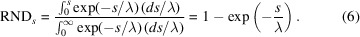

Inverting this expression we obtain

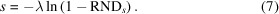




The pathlength is estimated using a computer-generated random number and equation (7), such that the calculated values for *s* obey the required Poisson distribution. The mean free path for plasmons can be measured experimentally from an electron energy-loss spectrum (Mendis, 2019 ▸). For uncorrelated phonons the TDS differential scattering cross section (σ_TDS_) for a single atom is (Pennycook & Jesson, 1991 ▸)

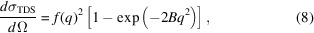

where Ω is the scattering solid angle, *f*(*q*) is the atom scattering factor and *B* is the Debye–Waller factor. The scattering vector magnitude is given by 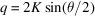 . The uncorrelated phonon mean free path λ_ph_ is then (Joy, 1995 ▸)

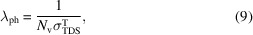

where *N*_v_ is the atomic number density and 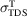 is the total TDS scattering cross section, obtained by integrating equation (8) over all solid angles.

The polar scattering angle θ can be similarly estimated using a uniform random number ( 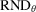 ) in the range [0, 1]. For plasmons the result is (Mendis, 2020*b* ▸)

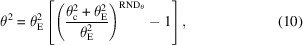

where θ_E_ and θ_c_ are the characteristic and critical angles, respectively, for plasmon scattering (Egerton, 1996 ▸). The equivalent random number for uncorrelated phonon scattering is

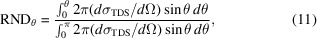

where we have made use of the relation 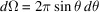 . Equation (11) does not have a straightforward analytical solution. Therefore, 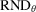 is first numerically evaluated for different values of θ. To estimate the phonon polar scattering angle, a uniform random number within the range [0, 1] is computer generated and the corresponding θ value obtained by interpolating the 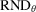 versus θ data points (Joy, 1995 ▸).

Low-energy phonon and plasmon excitations have a negligible effect on the incident electron wavenumber. For example, a 17 eV plasmon energy-loss event in silicon changes the wavenumber of a 200 kV electron by ∼0.004%. However, phonon and plasmon excitation do alter the electron trajectory [Fig. 1 ▸(*a*)], and for small scattering angles the ratio (*K*/*K*_n_) will be largely unchanged. Therefore, only the deviation parameter *s*_**g**_ in the structure matrix diagonal terms will vary as a result of inelastic scattering [equation (2)]. The new *s*_**g**_ value is easily determined from the electron wavevector **K**′ following inelastic scattering (Spence & Zuo, 1992 ▸),

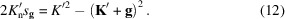




The structure matrix must contain a sufficiently large number of elements (diffracted beams) to accommodate the change in wavevector following inelastic scattering. For plasmons this is generally not an issue, due to the extremely small characteristic angle θ_E_ (*e.g.* 0.04 mrad for Si at 200 kV; Section 3). However, for some phonon events the scattering angle can be as large as 100 mrad [see Fig. 4(*a*)]. In practice, the number of beams is limited by the computational cost, so that some high-angle phonon events will not be simulated as accurately as low-angle inelastic scattering. Fig. 1 ▸(*b*) shows single inelastic scattering (plasmon or phonon) at a depth *s* in a specimen of thickness *t*. The elastic wavefunction 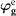 at a depth *s* is, from equation (4), given by

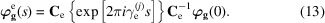

Here, **C**_e_ and 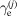 are the eigenvectors and eigenvalues, respectively, obtained by eigen decomposition of the structure matrix for elastic scattering [equation (3)]. Following inelastic scattering the wavefunction at the exit surface 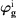 is

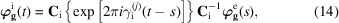

where **C**_i_ and 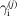 are the new eigenvectors and eigenvalues obtained by eigen decomposition of the structure matrix for inelastic scattering. The calculation must be repeated for multiple inelastic scattering configurations and the diffracted beam intensities added incoherently to give statistically accurate values, *i.e.*


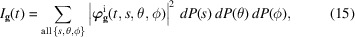

where *I*_**g**_(*t*) is the averaged diffracted beam intensity and 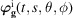 is the diffracted beam exit wavefunction for the {*s*, θ, ϕ} inelastic scattering configuration [equation (14)]. For uncorrelated phonon scattering, the *s*, θ and ϕ probability distributions are given by

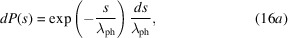




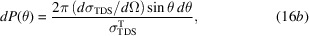




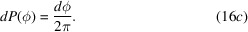


*dP*(*s*) is the probability of phonon scattering between *s* and *s* + *ds*, and similarly for *dP*(θ) and *dP*(ϕ). Equivalent expressions for correlated phonon scattering can be found in the supporting information. The probabilities for plasmon scattering are (Mendis, 2023*a* ▸)

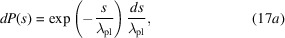




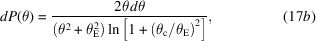




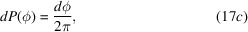

where λ_pl_ is the plasmon mean free path.

Evaluating equation (15) can be time consuming. An alternative way of determining *I*_**g**_(*t*) is to randomly select {*s*, θ, ϕ} inelastic configurations and calculate the average of 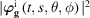 . The *s*, θ and ϕ values are chosen using computer-generated random numbers [*i.e.* equations (5), (7), (10) and (11)]. Since the configurations are chosen at random, further weighting by the probability distribution functions is not required.

The above discussion has focused on single inelastic scattering, although it is clear that the general methodology can be extended to multiple inelastic scattering as well. The polar and azimuthal scattering angles are defined with respect to the electron trajectory prior to inelastic scattering [Fig. 1 ▸(*a*)]. Therefore, where multiple inelastic scattering is involved, it is important to keep track of the electron wavevector with respect to a fixed frame of reference. This is easily achieved using Euler rotation matrices (Mendis, 2019 ▸).

## 3. Experimental and simulation methods

Silicon was used as a test specimen, since it is readily available as defect-free single crystals, and because its plasmon excitations have been well characterized (Mendis, 2019 ▸; Barthel *et al.*, 2019 ▸). An ion-beam polished Si [110] single-crystal sample was examined at 200 kV in a JEOL 2100F field-emission gun transmission electron microscope. Energy-filtered diffraction patterns were acquired with parallel-beam illumination and a Gatan Tridiem electron energy-loss spectroscopy (EELS) imaging filter equipped with an Ultrascan 1000 CCD camera. Four separate 10 eV energy windows, centred around the zero-loss peak, single (17 eV) and double (34 eV) plasmon energy and Si *L*_2,3_ core loss edge onset (100 eV), were used for energy filtering. The intensities of the diffraction patterns were within the linear response range of the CCD camera (see the supporting information). The EELS measured specimen thickness is 2.4 inelastic mean free paths, or ∼1990 Å for an inelastic mean free path value of ∼830 Å, calculated using the method of Malis *et al.* (1988 ▸).

Kirkland’s (2010 ▸) atom scattering factors were used to calculate the Bloch wave structure matrix. The simulation contained 625 zero-order Laue zone (ZOLZ) beams, including the unscattered beam. The atom scattering factor was only parameterized up to a 12 Å^−1^ scattering vector magnitude, and therefore this was set as the upper limit for calculating the total TDS scattering cross section 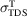 , as well as the upper limit of integration in the denominator for 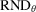 [equation (11)]. In practice, this truncation should have little effect on the accuracy, since the atom scattering factor has already decreased to an insignificant value at the cut-off. The r.m.s. thermal vibration of silicon atoms is 0.078 Å (Kirkland, 2010 ▸), which gives a Debye–Waller factor *B* of 0.12 Å^2^. The phonon mean free path at 200 kV, calculated using equation (9), is 7724 Å. This is a considerably larger value than the plasmon mean free path for silicon, which has been experimentally measured using EELS to be 1050 Å (Mendis, 2019 ▸). The longer mean free path for phonons is consistent with the results of Vos & Winkelmann (2019 ▸). The plasmon characteristic scattering angle is given by θ_E_ = Δ*E*/2*E*_o_, where Δ*E* is the plasmon energy loss and *E*_o_ is the primary beam energy (Egerton, 1996 ▸). For Si at 200 kV, θ_E_ = 0.04 mrad. The plasmon critical angle θ_c_ = 19.1 mrad is obtained by fitting simulation to experiment (Barthel *et al.*, 2019 ▸). θ_c_ has been corrected for the difference in acceleration voltage, *i.e.* 300 kV in Barthel *et al.* (2019 ▸), using the fact that the critical scattering vector *q*_c_ ≃ *K*θ_c_ is a constant. For energy-filtered diffraction, the number of plasmon events was chosen to match the desired energy window. For example, the diffraction pattern at 17 eV contains only single plasmon scattering, that at 34 eV contains double plasmon scattering *etc*. All the simulation results are for [110]-Si at 200 kV. The specimen thickness of 1990 Å is the same as the experimental value.

## 4. Results and discussion

### 4.1. Experimental results

**Figure 2:**
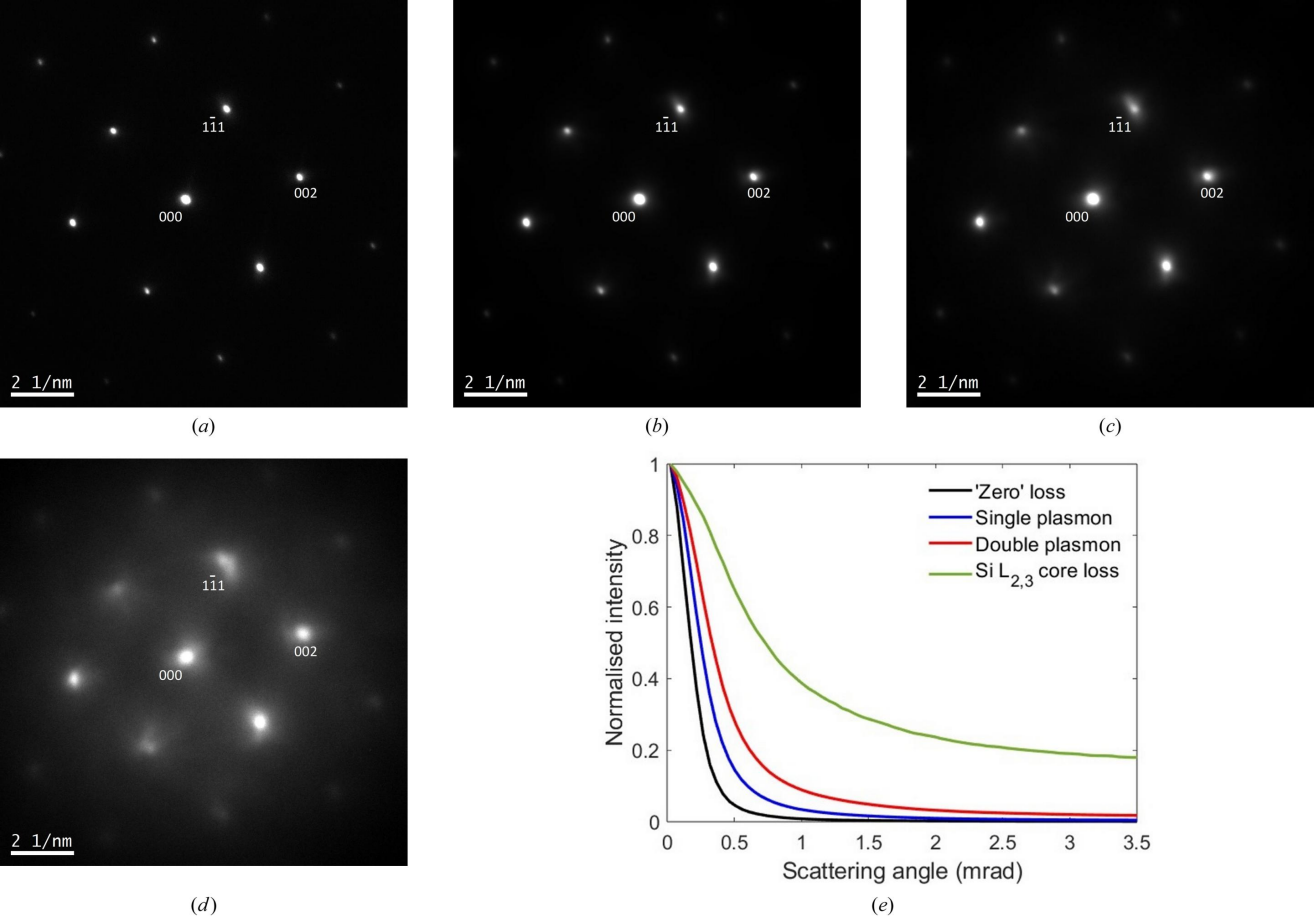
Experimental [110]-Si energy-filtered diffraction patterns at (*a*) ‘zero’ energy loss, (*b*) single plasmon (17 eV), (*c*) double plasmon (34 eV) and (*d*) Si *L*_2,3_ core loss edge onset (100 eV). (*e*) A superimposition of the average radial intensity profiles for the 000 unscattered beam in each diffraction pattern. The maximum intensity is normalized to unity.

Figs. 2 ▸(*a*)–2 ▸(*d*) show experimental energy-filtered [110]-Si selected-area diffraction patterns at ‘zero’ energy loss, single plasmon (17 eV), double plasmon (34 eV) and Si *L*_2,3_ core loss edge onset (100 eV). The 10 eV energy window used for acquisition cannot discriminate between phonon energy losses, and therefore all diffraction patterns contain some amount of thermal diffuse scattering.

Two trends are noticeable from the data. The first is that the diffracted beams become more diffuse with energy loss. This is clear from the radially averaged intensity profile for the 000 unscattered beam, shown in Fig. 2 ▸(*e*) for each energy-filtered diffraction pattern. The results are consistent with the observations of Eaglesham & Berger (1994 ▸), who reported a higher fraction of diffuse plasmon scattering outside the diffraction discs of a focused electron beam. Second, with increasing energy loss the intensity of the unscattered 000 beam decreases, resulting in an overall increase in the relative intensity of the Bragg reflections. Table 1 ▸ lists intensities for the 000, 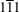 , 002 and 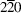 beams for each energy-filtered diffraction pattern. The intensity was extracted from a square region 300 pixels (*i.e.* 5.3 mrad scattering angle) wide, centred around the reflection of interest, which was large enough to include much of the diffuse scattering for that beam. The beam intensity was normalized by dividing by the total intensity for the entire diffraction pattern. Each set of Bragg beams contains multiple reflections that are symmetry related, *e.g.* there are four 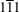 -type reflections in the [110] zone axis, from which the errors in Table 1 ▸ were estimated. The intensity ratios for Bragg-diffracted beams with respect to the 000 beam are also listed. Although there is a systematic decrease in the unscattered beam intensity with energy loss, the Bragg-diffracted beam intensities remain largely unchanged within the measurement error, so that overall there is a net increase in the Bragg beam intensity ratios (Table 1 ▸). For example, a single plasmon scattering event can change the 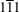 and 002 intensity ratio from ∼0.4 to 0.5, a relative increase of 25%. The higher energy loss Si *L*_2,3_ edge has an even greater effect on the intensity ratio, but the cross section for core loss excitation is comparatively small compared with plasmons (Egerton, 1996 ▸). Note that the decrease in 000 beam intensity is not due to a smaller scattering cross section at high energy loss, since the total intensity of each diffraction pattern is separately normalized to unity.

### 4.2. Simulation of diffuse scattering distributions

**Figure 3:**
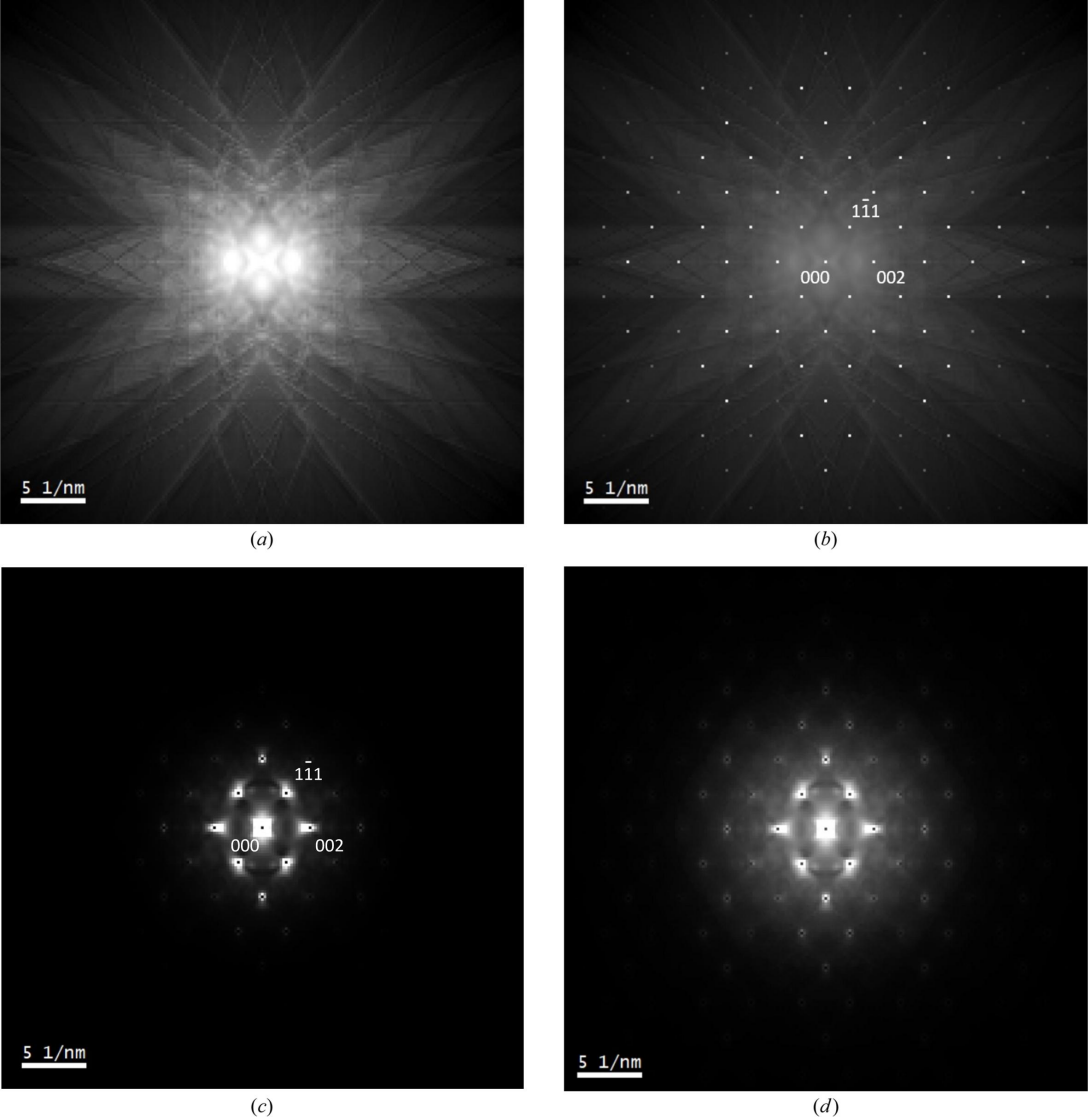
(*a*) Simulated thermal diffuse scattering in [110]-Si due to single phonon excitation. (*b*) The thermal diffuse scattered intensity superimposed on the elastic Bragg scattering. The figure is displayed using a square root intensity scale to highlight weak features in the diffraction pattern. (*c*) Simulated single plasmon diffuse scattering in [110]-Si. (*d*) The data from panel (*c*) displayed using a square root intensity scale to highlight weak features in the diffraction pattern.

Next we consider Bloch wave inelastic scattering simulations using the Monte Carlo method described in Section 2. The single-scatter phonon diffraction pattern for [110]-Si is shown in Fig. 3 ▸(*a*). The 1990 Å thick specimen was divided into slices 100 Å thick and the diffraction intensities calculated using equations (15) and (16*a* [Disp-formula fd16a])–(16*c* [Disp-formula fd16c]). The exit wavevector of the inelastically scattered electron beam was used to map the Bragg intensities in equation (15) to the corresponding pixels in the diffraction pattern. In other words, the deflection in electron trajectory during inelastic scattering rigidly shifts the Bragg diffraction pattern in reciprocal space. Since there is a continuous range of deflection angles, the net result is diffuse scattering. Rigid shifting of the diffraction pattern is theoretically justified for fully delocalized excitations, where it can be shown that the entire inelastic electron wavefunction shows particle-like behaviour (Mendis, 2020*a* ▸). Such conditions are likely to be satisfied for low-energy phonon and plasmon excitations in small unit cell materials such as silicon, since aloof beam EELS scattering measurements have recorded delocalization lengths of ∼10 nm for phonons (Krivanek *et al.*, 2014 ▸) and a few nanometres for plasmons (Zhou *et al.*, 2012 ▸). The assumption of ‘infinite’ delocalization, and hence rigid shifting of the electron diffraction pattern, may, however, not strictly hold for crystals with large unit cells. For highly localized excitations such as Compton scattering, intensity is largely transferred into the inelastic channel from only a single Bragg beam, leaving the other reflections unaltered (Mendis & Talmantaite, 2022 ▸; Mendis, 2023*b* ▸). The Compton scattering cross section is, however, relatively weak, and is therefore not considered here.

The angular distribution of the thermal diffuse scattering in Fig. 3 ▸(*a*) contains the expected Kikuchi bands and Kikuchi lines. This is a significant improvement over traditional Bloch wave calculations, which rely on a complex crystal potential to model phonon excitation, and therefore cannot provide any detailed information on the thermal diffuse scattering (Hirsch *et al.*, 1965 ▸). Higher-order Laue zone (HOLZ) rings and lines are, however, not present in Fig. 3 ▸(*a*), since only ZOLZ reflections were used in the calculations (Section 3). Although not a fundamental limitation, including HOLZ reflections in the Bloch wave calculation would significantly increase the simulation time, while having little effect on the ZOLZ beam intensities (Spence & Zuo, 1992 ▸). Sharp Bragg peaks are also not apparent in Fig. 3 ▸(*a*). This is because only incident electrons that have undergone exactly one phonon scattering event are simulated, which invariably results in a non-zero deflection angle [equation (16*b* [Disp-formula fd16b])]. Fig. 3 ▸(*b*) is the corresponding diffraction pattern that includes both single phonon scattering and elastic scattering (the square root of the intensity is plotted to reduce the dynamic range). The elastic contribution was calculated using equation (13), with *s* being set equal to the specimen thickness *t*. The elastic intensities were also weighted by 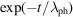 , the fraction of incident electrons not undergoing any phonon scattering, as determined by Poisson statistics. Fig. 3 ▸(*b*) is closer to the experimental ‘zero’ energy loss filtered diffraction pattern in this work [Fig. 2 ▸(*a*)], which includes both elastic and phonon losses due to the limited energy resolution. The only difference is the degree of phonon scattering, *i.e.* Fig. 3 ▸(*b*) is limited to a single phonon excitation, while the experiment will measure both single and multiple phonon scattering.

The simulated single plasmon scattered diffraction pattern is shown in Fig. 3 ▸(*c*). The 1990 Å thick specimen was divided into slices 100 Å thick and the diffraction intensities calculated using equations (15) and (17*a* [Disp-formula fd17a])–(17*c* [Disp-formula fd17c]). Due to the plasmon scattering angle, there is zero intensity at the exact Bragg positions, but this is not observed experimentally [Fig. 2 ▸(*b*)]. Unlike the phonon case, the missing Bragg intensity cannot be explained by purely elastic scattered electrons, since Fig. 2 ▸(*b*) was acquired at the single plasmon energy-loss value of 17 eV. Thus the intensity dip must be a real feature in the energy-filtered diffraction pattern. In practice, however, it would be difficult to observe this feature experimentally, due to the extremely small characteristic angle for plasmon scattering (0.04 mrad). Such a narrow intensity decrease is likely to be ‘smeared out’ by the point spread function of the detector, any imperfections in the crystal, and the incident-beam convergence angle. The intensity dip is only visible in Fig. 3 ▸(*b*) due to the finite size (0.5 mrad) of the pixels in the simulation, and because a perfectly parallel incident beam and ideal detector are assumed. The simulation also shows broad ‘halos’ around the Bragg positions, which are indeed observed experimentally [Figs. 2 ▸(*b*) and 2 ▸(*e*)]. Note that similar halos were not observed for thermal diffuse scattering [Figs. 2 ▸(*a*), 2 ▸(*e*) and 3 ▸(*a*)]. This is easily explained by examining equation (8), which indicates that the uncorrelated phonon intensity decreases to zero at small scattering vector magnitudes *q*.

**Figure 4:**
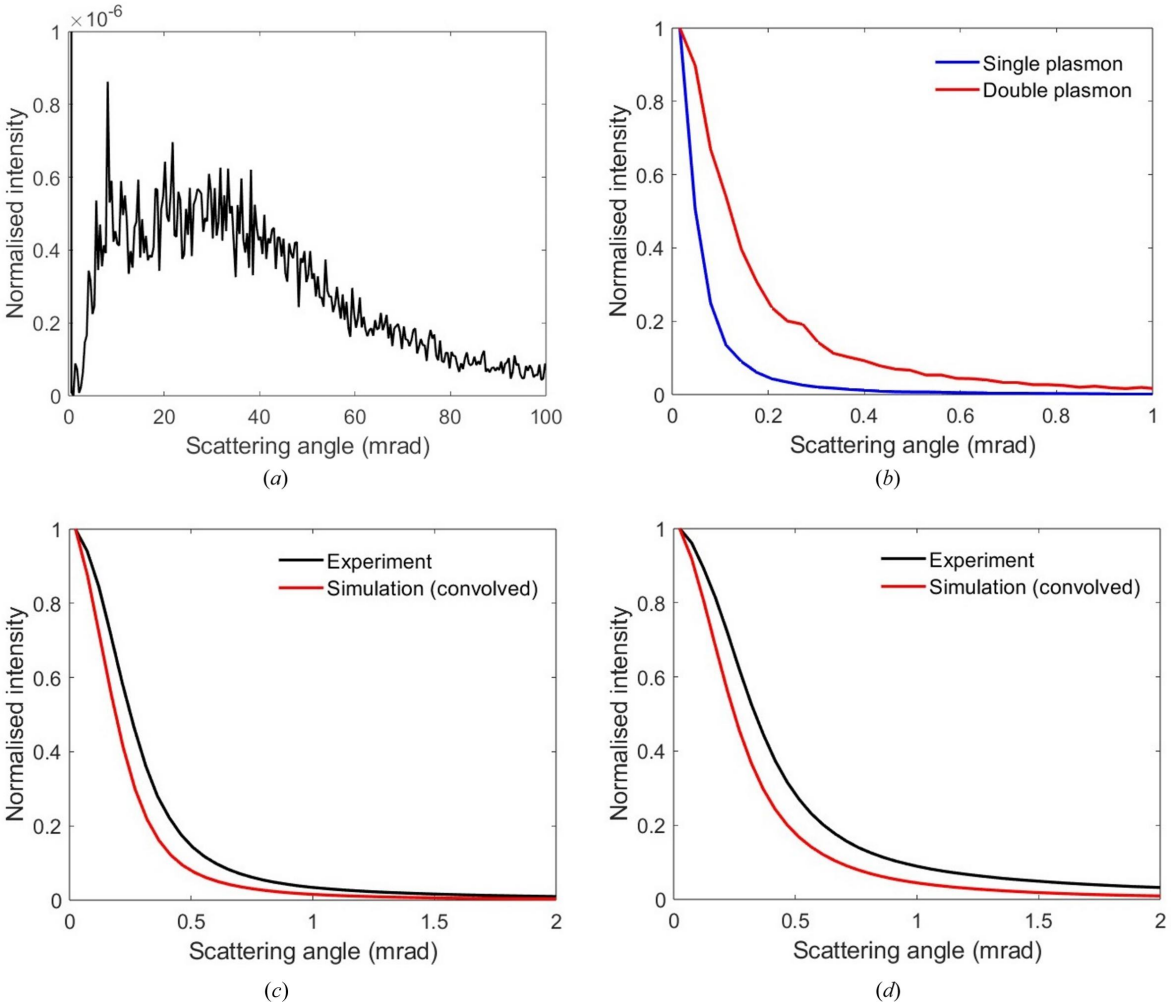
(*a*) The simulated average radial intensity profile for the elastic and thermal diffuse scattering around the 000 beam. (*b*) A superimposition of the simulated average radial intensity profiles for single and double plasmon scattering. (*c*), (*d*) Comparisons of simulated and experimental radial intensity profiles for (*c*) single and (*d*) double plasmon scattering. The simulation was convolved with the ‘zero’ loss average radial intensity profile of Fig. 2(*e*) for a direct comparison. In all of the profiles the maximum intensity has been normalized to unity [for panel (*a*) the maximum is the elastic peak at zero scattering angle, which is shown truncated].

Fig. 3 ▸(*d*) shows the plasmon diffuse scattering plotted on a square root intensity scale, in order to highlight weak features in the diffraction pattern. The plasmon diffuse scattering is free of Kikuchi band contrast, unlike that for phonons [Fig. 3 ▸(*a*)]. Kikuchi bands are formed by the inelastic diffuse intensity that has been deflected towards crystal planes at the Bragg angle, so that strong diffraction is possible (Hirsch *et al.*, 1965 ▸). This is easily achieved with phonons, which can have large scattering angles [see Fig. 4 ▸(*a*)], but for plasmons the diffuse intensity has a more narrow distribution due to the small characteristic angle θ_E_. The fraction of plasmon scattered electrons at the Bragg angle is therefore small, so that the contrast of any Kikuchi bands will be weak. For example, at the 4 mrad Bragg angle for the 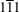 reflection in silicon (200 kV electron beam), the plasmon differential scattering cross section has decreased to 0.01% of its maximum value, assuming a Lorentzian dependence (Egerton, 1996 ▸).

To reproduce the plasmon energy-filtered results in Figs. 2 ▸(*b*) and 2 ▸(*c*), both plasmon and phonon scattering must be included in the simulation. Although the number of plasmon events is fixed by the energy loss, the experimental diffraction patterns can still contain multiple phonon scattering, since a 10 eV window was used for the energy filtering. Therefore, in the simulations phonon events were selected at random, while keeping the number of plasmon excitations fixed. For example, consider simulating the single plasmon energy-filtered diffraction pattern. First the plasmon scattering depth *s* was estimated using a computer-generated random number and equation (7). Phonon excitation can occur prior to plasmon scattering (*i.e.* at depths smaller than *s*), as well as after the plasmon event (*i.e.* at depths larger than *s*). For the former, phonon scattering depths were estimated using equation (7); multiple phonon events are allowed, as long as the cumulative scattering path length does not exceed *s* (there are, however, no phonon events if the first scattering depth is larger than *s*). A similar procedure was used to simulate phonon excitation following the plasmon event, as the electron beam propagates to the specimen exit surface. Due to inelastic scattering, there will be a change in the electron wavevector at the specimen exit surface, which gives rise to the diffuse background intensity in the diffraction pattern. Since inelastic events are chosen at random, multiple iterations of electron-beam propagation through the specimen must be incoherently averaged to produce a numerically converged result (in this work 50 000 iterations were used). Double plasmon energy-filtered diffraction patterns can also be simulated in a similar manner, except that there are now two plasmon scattering events interspersed with multiple phonon excitation.

Fig. 4 ▸(*a*) is the simulated average radial intensity profile for the 000 unscattered beam at ‘zero’ loss energy filtering. The simulation contains elastic and phonon scattered electrons, but no plasmon losses. The peak intensity of the 000 beam has been normalized to unity. The thermal diffuse scattering is six orders of magnitude smaller than the 000 beam peak intensity and shows a broad maximum between 10 and 40 mrad in scattering angle. The overall shape is similar to TDS scattering from a single atom (Pennycook & Jesson, 1991 ▸) and has its origin in equation (8). The simulated average radial intensity profiles for the 000 unscattered beam at single and double plasmon loss energy filtering are shown superimposed in Fig. 4 ▸(*b*). The plasmon diffuse intensity is more concentrated around the 000 beam, and although TDS is also present it is too weak to be seen in the full intensity scale of Fig. 4 ▸(*b*). Figs. 4 ▸(*c*) and 4 ▸(*d*) compare the experimental and simulated radial intensity profiles for single and double plasmon energy losses, respectively. The experimental data were obtained from Fig. 2 ▸(*e*). The simulated profiles [Fig. 4 ▸(*b*)] were convolved with the experimental ‘zero’ loss profile [Fig. 2 ▸(*e*)], which is assumed to be representative of the point spread function of the detector, as well as any crystal imperfections and beam convergence [note that for a perfect crystal the main peak of the ‘zero’ loss profile should be a delta function; Fig. 4 ▸(*a*)]. Even after convolution, the simulated profiles are slightly narrower than the experimental ones, which may be due to errors in the plasmon scattering parameters (*e.g.* the characteristic angle θ_E_ is too small) or because a Lorentz model does not accurately describe the plasmon differential cross section (Egerton, 1996 ▸). A direct comparison of the convolved simulated profiles with all experimental profiles in Fig. 2 ▸(*e*) can be found in the supporting information.

### 4.3. Energy-filtered diffracted beam intensities

Table 2 ▸ lists the simulated beam intensities and Bragg beam intensity ratios for ‘zero’ loss, single and double plasmon energy-filtered diffraction patterns. The equivalent result at the Si *L*_2,3_ core loss edge would require a transition matrix element calculation (Maslen & Rossouw, 1984 ▸; Allen *et al.*, 2015 ▸), which is not attempted here. The intensity of the 000 beam and Bragg beam intensity ratios in Table 2 ▸ do not agree with the corresponding experimental values (Table 1 ▸). This could be partly due to inaccuracies in modelling inelastic scattering, as is evident from Figs. 4 ▸(*c*) and 4 ▸(*d*). Often there is also poor agreement between (elastic scattering) simulations and experimental ‘static’ electron diffraction patterns, acquired at a fixed specimen orientation and incident wavevector (Klar *et al.*, 2023 ▸). The discrepancy has been attributed to complex specimen shapes, sample bending, crystal mosaicity *etc*. Similar artefacts may be present in our measurements as well. Although some of the simulated values may not be in perfect agreement with the experimental results, they nevertheless capture the important trends, *i.e.* the unscattered 000 beam intensity systematically decreases with energy loss, leading to an overall increase in the Bragg beam intensity ratios.

Previous experimental work on energy-filtered diffraction has shown that Bragg peaks can be suppressed at large energy losses Δ*E* (Egerton, 1996 ▸). This occurs when the characteristic scattering angle θ_E_ = Δ*E*/2*E*_o_ becomes larger than the Bragg angle θ_B_. In this work, θ_E_ for plasmon scattering (0.04 mrad) is two orders of magnitude smaller than the innermost Bragg angle (4 mrad), so that Bragg peaks are clearly visible in the energy-filtered diffraction patterns [Figs. 2 ▸(*b*) and 2 ▸(*c*)]. Because 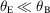 , there should be very little change in the intensity for a given Bragg reflection upon plasmon scattering. However, this need not be the case for the unscattered beam, since the cumulative effect of small amounts of intensity transfer to multiple Bragg beams can potentially lead to a noticeable change in the 000 beam intensity. This is consistent with the data in Tables 1 ▸ and 2 ▸. It could be argued that the decrease in 000 beam intensity with energy filtering is due to increased diffuse scattering, rather than subtle changes in the Bragg diffraction conditions. This can nevertheless be ruled out, since the experimental and simulated intensities in Tables 1 ▸ and 2 ▸ already include the diffuse scattering around the beam of interest. For example, the experimental intensities were extracted from a square region (5.3 mrad dimension) wider than the radial intensity profiles for single and double plasmon scattering [Fig. 2 ▸(*e*)]. The intensities in Tables 1 ▸ and 2 ▸ are normalized with respect to the total diffraction pattern intensity, so that changes in the inelastic scattering cross section with energy loss also have no effect on the results.

The azimuthal angle ϕ for plasmon scattering is purely random, and therefore some plasmon events will deflect the incident beam closer to the Bragg angle, thereby increasing the intensity for that reflection, while other events will have the opposite effect. Given the random nature of this process it is perhaps surprising that the 000 beam intensity decreases with energy loss, rather than staying roughly constant. Simulations at other specimen thicknesses (*i.e.* 500, 1000, 1500 and 2500 Å) show a similar trend (see the supporting information), although it is not known if this behaviour is universally valid. The scattering is somewhat analogous to precession electron diffraction, where the incident-beam wavevector can lie anywhere along a hollow cone (Vincent & Midgley, 1994 ▸; Midgley & Eggeman, 2015 ▸). The plasmon ‘precession’ angle is, however, very small, *i.e.* of the order of θ_E_. Elastic scattering simulations suggest that for small precession angles the 000 beam intensity is generally weaker than for normal beam incidence (see the supporting information). Elastic precession diffraction simulations were therefore performed with the precession angle set to various multiples of the plasmon characteristic scattering angle (*i.e.* θ_E_, 5θ_E_ and 10θ_E_). The azimuthal angle of the incident wavevector was sampled using 500 uniformly spaced points along the precession cone. In Table 3 ▸ the precession intensities are compared against the elastic scattered intensities for normal beam incidence. There is a slight decrease in the 000 beam intensity with increasing precession angle, which results in the Bragg intensity ratios also increasing. The changes in 000 beam intensity are, however, much smaller than those in Table 2 ▸. This is to be expected, since elastic precession diffraction is not a true like-for-like comparison with inelastic scattering in a real sample, which contains both plasmon and (potentially multiple) phonon scattering. The phonon scattering angle [Fig. 4 ▸(*a*)] can also be much larger than the precession angles in Table 3 ▸, which are based on θ_E_. Nevertheless, it does seem to provide a plausible explanation for the changes in beam intensities observed at different energy losses, although a more rigorous treatment is required to confirm its validity.

## 5. Summary

Inelastic scattering is often unavoidable in electron diffraction. For example, even if the specimen is only 100 Å thick, ∼10% of electrons will still undergo inelastic scattering, assuming Poisson statistics and an inelastic mean free path of 1000 Å. In this work, a combined Bloch wave–Monte Carlo simulation method is proposed to model Bragg diffraction in the presence of phonon and plasmon excitation, the two inelastic scattering mechanisms with the largest cross sections. With this method, it is possible to simulate thermal diffuse scattering accurately, including Kikuchi bands and Kikuchi lines. This is a significant improvement over phenomenological Bloch wave TDS calculations, where the imaginary term in the complex crystal potential depletes the electron-beam intensity as it propagates through the specimen, and where no information on the angular distribution of the TDS intensity is available. The plasmon diffuse intensity, on the other hand, shows no Kikuchi band contrast and is sharply peaked around the unscattered and Bragg beam reflections. This is due to the extremely small characteristic scattering angle for plasmons.

Experimental energy-filtered diffraction patterns in the low-loss EELS regime for [110]-Si showed that there was a systematic decrease in the 000 beam intensity with increasing energy loss, even after adjusting for differences in the inelastic scattering cross sections. Any changes to the Bragg beam intensities were, however, within the measurement error. Thus, the intensity ratio of a Bragg beam with respect to the unscattered beam increases with energy loss. Simulations are able to reproduce these trends across a wide range of specimen thicknesses. It is speculated that the decrease in 000 beam intensity is due to a precession effect caused by the random change in azimuthal angle of the incident beam during inelastic scattering. Here the dominant inelastic scattering mechanism is plasmon excitation, which has a much shorter mean free path than phonons (*e.g.* 1050 Å versus 7724 Å for Si at 200 kV). The precession angle is therefore of the order of the plasmon characteristic scattering angle θ_E_. The precession effect results in a net intensity transfer from the 000 beam to the inner Bragg reflections. The intensity change for a single Bragg reflection will be small, however, since θ_E_ is considerably smaller than the Bragg angle.

It is possible to speculate how low-loss inelastic scattering might influence crystal structure refinement. Typically, the unscattered beam is ignored and only the relative intensities of the Bragg beams are used to solve the crystal structure. For the low energy loss regime, however, the relative intensity ratios between the diffracted beams are approximately constant (Table 1 ▸). Therefore, inelastic scattering should not have any significant effect on crystal structure refinement, which directly contradicts experimental observations of improved results obtained with energy filtering (Gemmi & Oleynikov, 2013 ▸; Eggeman *et al.*, 2013 ▸; Yang *et al.*, 2022 ▸). According to Yang *et al.* (2022 ▸), energy filtering produces less background and sharper diffraction spots, which makes it easier to extract the diffracted beam intensities. Eggeman *et al.* (2013 ▸) have shown that the inelastic background has a larger influence on weak reflections, resulting in higher *R* values. In our analysis, however, the background was not subtracted from the diffracted beam intensities (see Section 3). This is especially important for higher energy losses, where a larger fraction of the diffracted beam intensity appears as diffuse scattering [Figs. 2 ▸(*e*) and 4 ▸(*b*)]. Background subtraction in unfiltered diffraction patterns could therefore result in higher *R* values than with no background subtraction. On energy filtering the diffuse scattering is largely removed, so that any background subtraction will introduce fewer artefacts, resulting in smaller *R* values. Phonon losses will nevertheless still be present even after energy filtering. Table 2 ▸ includes the beam intensities for a fully elastic calculation, which are similar to the values obtained for a ‘zero’ loss simulation that includes both elastic and phonon scattering. This suggests that a fully elastic calculation can be used to refine energy-filtered diffraction data without introducing any further artefacts. Finally, it should be noted that our conclusions are only valid for low energy loss excitations, *i.e.* phonons and plasmons. If the specimen is very thick, core loss edges and Compton scattering become non-negligible. The latter is also present as diffuse scattering over a large section of reciprocal space. The role of these high energy loss events on crystal structure refinement is unknown.

The computer code for this work is available open access from the Durham University research data repository (DOI: https://doi.org/10.15128/r2x920fw895).

## 6. Related literature

For further literature related to the supporting information, see Martin *et al.* (2009 ▸) and Mendis (2022 ▸).

## Supplementary Material

Additional background, tables and figures. DOI: 10.1107/S2053273323010690/tw5007sup1.pdf

## Figures and Tables

**Table 1:** Experimental beam intensities for ‘zero’ loss, single plasmon (17 eV), double plasmon (34 eV) and Si *L*_2,3_ core loss (100 eV) energy-filtered diffraction patterns The total intensity for the diffraction pattern at a given energy loss is normalized to unity. The intensity ratios for a given Bragg beam are calculated with respect to the unscattered 000 beam.

	000 intensity	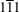 intensity	002 intensity	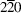 intensity
‘Zero’ loss	0.16	0.07 ± 0.03	0.06 ± 0.01	0.03 ± 0.01
Single plasmon	0.13	0.07 ± 0.02	0.07 ± 0.01	0.02 ± (<0.01)
Double plasmon	0.10	0.06 ± 0.02	0.06 ± 0.01	0.02 ± (<0.01)
Si *L*_2,3_ core loss	0.05	0.04 ± 0.01	0.04 ± (<0.01)	0.02 ± (<0.01)
				
		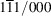 ratio	002/000 ratio	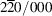 ratio
‘Zero’ loss		0.43 ± 0.16	0.40 ± 0.04	0.16 ± 0.04
Single plasmon		0.51 ± 0.16	0.51 ± 0.05	0.18 ± 0.01
Double plasmon		0.60 ± 0.16	0.60 ± 0.08	0.22 ± (<0.01)
Si *L*_2,3_ core loss		0.82 ± 0.12	0.77 ± 0.09	0.45 ± 0.03

**Table 2:** Simulated beam intensities for ‘zero’ loss, single plasmon and double plasmon energy-filtered diffraction patterns (50 000 iterations) The ‘zero’ loss simulation contains phonon events but no plasmons. Also shown are the results for a fully elastic calculation (*i.e.* no inelastic events). The total intensity for the diffraction pattern at a given energy loss is normalized to unity. The intensity ratios for a given Bragg beam are calculated with respect to the unscattered 000 beam.

	000 intensity	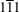 intensity	002 intensity	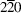 intensity
‘Zero’ loss	0.48	0.07	<0.01	0.04
Single plasmon	0.30	0.09	0.03	0.05
Double plasmon	0.21	0.08	0.04	0.05
Fully elastic	0.46	0.09	<0.01	0.04
				
		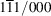 ratio	002/000 ratio	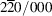 ratio
‘Zero’ loss		0.16	<0.01	0.08
Single plasmon		0.29	0.09	0.16
Double plasmon		0.38	0.18	0.24
Fully elastic		0.19	<0.01	0.09

**Table 3:** Simulated intensities for normal incidence and precession angles of θ_E_, 5θ_E_ and 10θ_E_, where θ_E_ is the plasmon characteristic scattering angle (0.04 mrad) The precession calculations included 500 uniformly spaced incident wavevectors along the precession cone. The intensity ratios for a given Bragg beam are calculated with respect to the unscattered 000 beam. The results do not include any inelastic scattering. In each case the total intensity of the diffraction pattern was normalized to unity.

	000 intensity	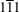 intensity	002 intensity	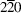 intensity
Normal incidence	0.46	0.09	<0.01	0.04
Precession (θ_E_)	0.46	0.09	<0.01	0.04
Precession (5θ_E_)	0.45	0.09	<0.01	0.04
Precession (10θ_E_)	0.44	0.08	<0.01	0.05
				
		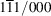 ratio	002/000 ratio	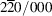 ratio
Normal incidence		0.19	<0.01	0.09
Precession (θ_E_)		0.19	<0.01	0.09
Precession (5θ_E_)		0.19	<0.01	0.10
Precession (10θ_E_)		0.20	0.01	0.11
