# Supplementary material for: Modelling dynamical 3D electron diffraction intensities. II. The role of inelastic scattering
Source: Acta Crystallogr A Found Adv. 2024 Jan 25;80(Pt 2):178–88. doi: 10.1107/S2053273323010690 (PMC10913673; doi:10.1107/S2053273323010690)
Supplement: Supplementary file 1 [file a-80-00178-sup1.pdf]

FOUNDATIONS
ADVANCES

Volume 80 (2024)

Supporting information for article:

Modelling dynamical 3D electron diffraction intensities. II. The role of inelastic scattering

Budhika Mendis

(1) Monte Carlo probabilities for correlated phonon scattering

Equations 16a-16c are the Monte Carlo probabilities for uncorrelated phonons. Here equivalent expressions are derived for correlated phonons. Since momentum is conserved during inelastic scattering, a phonon with wavevector $\mathbf{k}$ will scatter along the scattering vector $\mathbf{q} = -\mathbf{k}$, with cross-section $\sigma_{\mathbf{k}}$. Let $\mathbf{q}_{\perp}$ be the component of $\mathbf{q}$ that is perpendicular to the direction of the incident electron. Instead of polar and azimuthal scattering angles θ and ϕ, we can use $\mathbf{q}_{\perp}$ to represent the direction of the incident electron following inelastic scattering. The Monte Carlo probabilities are then:

$$dP(s) = \exp\left(-\frac{s}{\lambda_{\mathrm{ph}}}\right)\frac{ds}{\lambda_{\mathrm{ph}}}$$

… (S1)

$$dP(\mathbf{q}_{\perp}) = \frac{\sum_{\mathbf{k}} \sigma_{\mathbf{k}} \delta(\mathbf{k}_{\perp} + \mathbf{q}_{\perp})}{\sum_{\mathbf{k}} \sigma_{\mathbf{k}}}$$

… (S2)

where the summation in Equation S2 is over all phonon wavevectors $\mathbf{k}$. $\mathbf{k}_{\perp}$ is the phonon wavevector component perpendicular to the incident electron direction. The delta function in the numerator ensures momentum conservation. Following Equation 9 the phonon mean free path is:

$$\lambda_{\mathrm{ph}} = \frac{1}{N_v(\sum_{\mathbf{k}} \sigma_{\mathbf{k}})}$$

… (S3)

The phonon scattering cross-sections $\sigma_{\mathbf{k}}$ can be derived quantum mechanically; see for example, Martin *et al. Phys. Rev. B* **80** (2009) 024308 or Supplementary Information for Mendis, *Ultramicroscopy* **239** (2022) 113548.

(2) Linearity of CCD camera

The energy filtered diffraction patterns in Figure 2 (main text) were acquired on a Gatan Ultrascan 1000 CCD camera with no binning (i.e. 2048 x 2048 pixels). To test its linearity, the

CCD was evenly illuminated with no specimen in the field of view and images acquired at different exposure times. Dark subtraction and gain correction were applied to the images. Figure S1 shows the average counts per pixel as a function of exposure time.

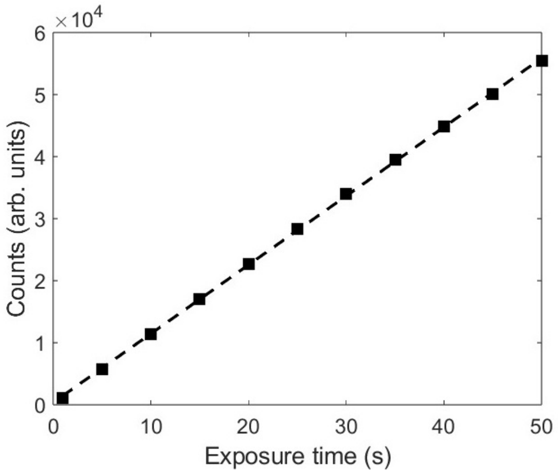

Figure S1: Average number of counts per pixel as a function of exposure time for a Gatan Ultrascan 1000 CCD camera. The dashed line is the best fit straight line to the data points.

The imaging is linear up to the maximum number of counts recorded (~56,000), although the first hint that the detector was approaching saturation was observed at ~45,000 counts. On the other hand, the maximum number of counts in the energy filtered diffraction patterns varied between ~2,000 to ~35,000 counts, well below saturation and within the linear response region of the CCD.

(3) Comparison of simulated and experimental diffuse scattering profiles

Figure S2 shows the experimental energy filtered diffuse scattering profiles in Figure 2e superimposed with the convolved simulated profiles for single and double plasmon scattering in Figures 4c and 4d respectively.

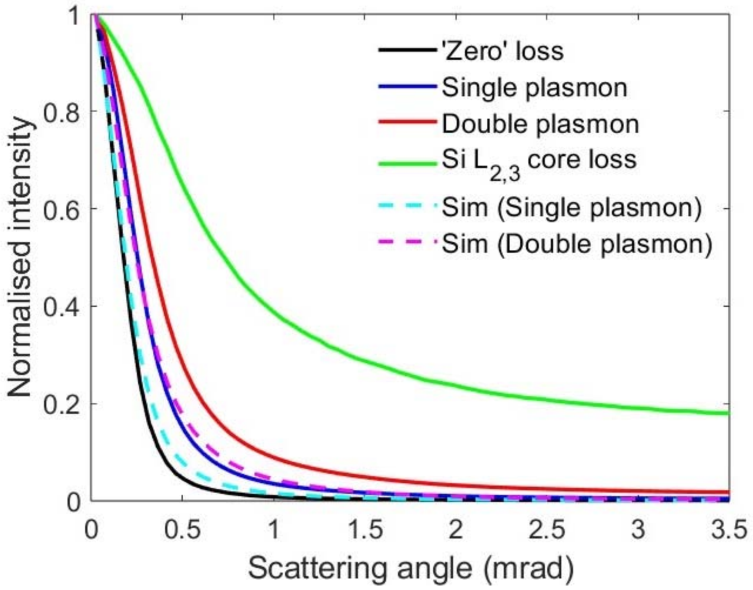

Figure S2: Experimental and simulated diffuse scattering profiles as a function of energy loss. The convolved simulated profiles for single and double plasmon scattering are labelled ‘Sim (Single Plasmon)’ and ‘Sim (Double Plasmon)’ respectively.

(4) Energy filtered [110]-Si beam intensities at different specimen thicknesses

Tables S1, S2, S3 and S4 list simulated beam intensities and Bragg intensity ratios for [110]-Si at specimen thicknesses of 500, 1000, 1500 and 2500 Å respectively. In each case, results for ‘zero’ energy loss, as well as single and double plasmon energy filtering are presented. The data confirm the precession effect at the different specimen thicknesses, i.e. 000 beam intensity decreases with energy loss, while the Bragg intensity ratios increase. Note that for the 2500 Å thick sample the decrease in 000 beam intensity for double plasmon loss is not apparent due to the rounding of numbers, i.e. to four significant figures the 000 beam intensity is 0.0829 for single plasmon and 0.0776 for double plasmon.

Table S1: Simulated beam intensities for ‘zero’ loss, single plasmon and double plasmon energy filtered diffraction patterns (50,000 iterations) in 500 Å thick, [110]-Si. The intensity ratios for a given Bragg beam are calculated with respect to the unscattered 000 beam.

	000 intensity	$1\bar{1}1$ intensity	002 intensity	$2\bar{2}0$ intensity
‘Zero’ loss	0.48	0.05	0.11	0.02
Single plasmon	0.40	0.06	0.09	0.03
Double plasmon	0.34	0.06	0.08	0.03
		$1\bar{1}1$/000 ratio	002/000 ratio	$2\bar{2}0$/000 ratio
‘Zero’ loss		0.11	0.23	0.04
Single plasmon		0.21	0.31	0.09
Double plasmon		0.31	0.37	0.16

Table S2: Simulated beam intensities for ‘zero’ loss, single plasmon and double plasmon energy filtered diffraction patterns (50,000 iterations) in 1000 Å thick, [110]-Si. The intensity ratios for a given Bragg beam are calculated with respect to the unscattered 000 beam.

	000 intensity	$1\bar{1}1$ intensity	002 intensity	$2\bar{2}0$ intensity
‘Zero’ loss	0.19	0.06	0.23	0.02
Single plasmon	0.13	0.07	0.18	0.03
Double plasmon	0.11	0.08	0.14	0.04
		$1\bar{1}1$/000 ratio	002/000 ratio	$2\bar{2}0$/000 ratio
‘Zero’ loss		0.12	0.48	0.04
Single plasmon		0.24	0.61	0.09
Double plasmon		0.36	0.65	0.17

Table S3: Simulated beam intensities for ‘zero’ loss, single plasmon and double plasmon energy filtered diffraction patterns (50,000 iterations) in 1500 Å thick, [110]-Si. The intensity ratios for a given Bragg beam are calculated with respect to the unscattered 000 beam.

	000 intensity	$1\bar{1}1$ intensity	002 intensity	$2\bar{2}0$ intensity
‘Zero’ loss	0.61	0.01	0.11	0.01
Single plasmon	0.44	0.03	0.11	0.02
Double plasmon	0.31	0.04	0.09	0.03
		$1\bar{1}1$/000 ratio	002/000 ratio	$2\bar{2}0$/000 ratio
‘Zero’ loss		0.02	0.23	0.02
Single plasmon		0.10	0.36	0.07
Double plasmon		0.20	0.43	0.14

Table S4: Simulated beam intensities for ‘zero’ loss, single plasmon and double plasmon energy filtered diffraction patterns (50,000 iterations) in 2500 Å thick, [110]-Si. The intensity ratios for a given Bragg beam are calculated with respect to the unscattered 000 beam.

	000 intensity	$1\bar{1}1$ intensity	002 intensity	$2\bar{2}0$ intensity
‘Zero’ loss	0.18	0.10	0.06	0.05
Single plasmon	0.08	0.11	0.07	0.06
Double plasmon	0.08	0.10	0.06	0.05
		$1\bar{1}1$/000 ratio	002/000 ratio	$2\bar{2}0$/000 ratio
‘Zero’ loss		0.20	0.13	0.11
Single plasmon		0.36	0.23	0.20
Double plasmon		0.46	0.29	0.26

(5) 000 beam intensity pendellösung in precession electron diffraction

The 000 unscattered beam intensity pendellösung was calculated for several precession angles (i.e. 0.1°, 0.5°, 1.0° and 2.0°) in [110]-Si and compared with the corresponding pendellösung at normal beam incidence. Only elastic scattering was considered, and 500 uniformly spaced azimuthal angles of the precession cone were incoherently averaged. The results are plotted in Figure S3. For large precession angles (e.g. 2.0°) the 000 beam intensity is generally higher in precession electron diffraction (PED), presumably because the scattering is more kinematic, and there is consequently less intensity transfer from the 000 beam into Bragg diffracted beams. For smaller precession angles, such as 0.1°, the pendellösung for PED and normal incidence begin to converge as expected. However, the 000 beam intensity for the former is generally lower. Note that small precession angles are equivalent to low energy inelastic scattering over random azimuthal angles.

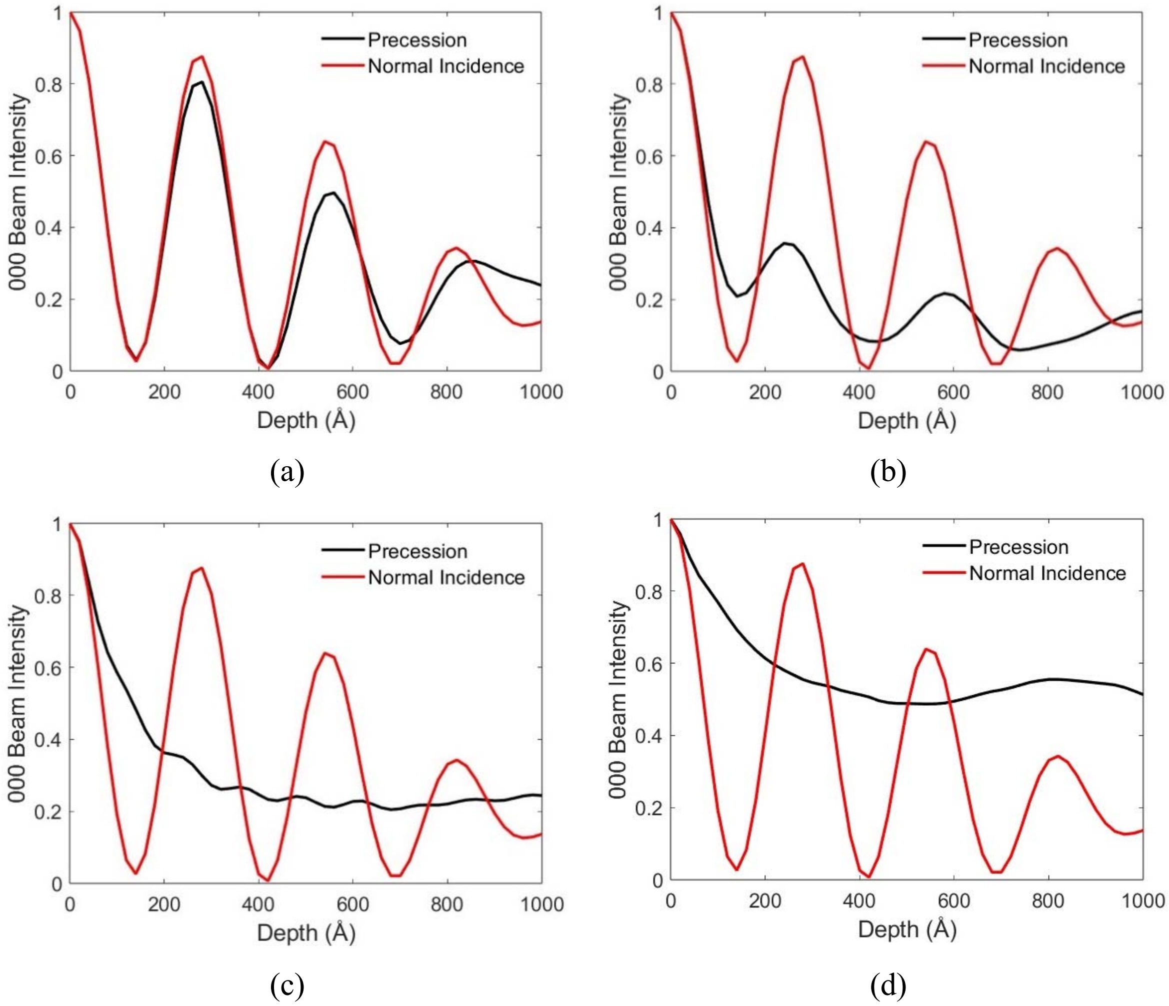

Figure S3: 000 beam intensity pendellösung for precession electron diffraction and normal beam incidence. The precession angle is (a) 0.1º, (b) 0.5º, (c) 1.0º and (d) 2.0º. Only elastic scattering is considered.
